# Eating Disorders and Suicidal Behaviors in Adolescents with Major Depression: Insights from the US Hospitals

**DOI:** 10.3390/bs11050078

**Published:** 2021-05-19

**Authors:** Rikinkumar S. Patel, Tanya Machado, William E. Tankersley

**Affiliations:** 1 Department of Psychiatry, Griffin Memorial Hospital, Norman, OK 73071, USA; wtankersley@odmhsas.org; 2 Department of Psychiatry and Behavioral Sciences, Oklahoma State University, Tulsa, OK 74106, USA; 3 Father Muller Medical College, Mangalore 575002, India; tanya.nicole94@gmail.com

**Keywords:** eating disorders, bulimia nervosa, anorexia nervosa, nationwide inpatient sample, suicide ideations, suicidal attempts

## Abstract

Objective: To evaluate the odds of association between suicidal ideation and/or attempt with comorbid eating disorders in adolescents with major depressive disorder (MDD). Methods: We conducted a cross-sectional study and included 122,020 adolescents with a primary diagnosis of MDD from the nationwide inpatient sample (NIS, 2012–2014). They were sub-grouped by a comorbid diagnosis of eating disorders (N = 1675). We calculated the adjusted odds ratio (aOR) using a logistic regression model with demographic confounders for associations of eating disorders with suicidal ideation and attempt. Results: Suicidal ideations were seen in a higher proportion of adolescents with eating disorders (46.3% vs. 14.2% in those without eating disorders). On the contrary, a low proportion of adolescents with eating disorders had suicidal attempts (0.9% vs. 39.4% in those without eating disorders). Overall, eating disorders were associated with higher odds for suicidal ideations (aOR 5.36, 95% CI 4.82–5.97) compared to those without eating disorders, but with lower odds of suicidal attempt (aOR 0.02, 95% CI 0.01–0.03). Conclusions: Adolescents with MDD and comorbid eating disorders had five-times increased odds of suicidal ideations but lower odds of a suicide attempt. Self-harm/injurious behaviors are early signs of suicidal ideations in these patients. A collaborative care model is required for the screening, early diagnosis, and management of adolescents with eating disorders to improve their quality of life.

## 1. Introduction

Children between the ages of 5 and 11 have a suicide rate of 0.17 per 100,000 individuals. Among adolescents, there is an increased suicide rate of 5.18 per 100,000 individuals in adolescents [1]. Adolescent boys (15–19 years old) have a three-times higher risk of fatal suicide attempts than girls of the same age. However, the rate of non-fatal suicide attempts is twice as high among girls compared to boys [2].

The prevalence of eating disorders in adolescents is 0.3% for anorexia nervosa and 0.9% for bulimia nervosa and is lower than that reported in the adult population [3]. Around 23–48% of adolescents with eating disorders have comorbid depression, with a higher prevalence rate seen in patients with bulimia nervosa (50%) than those with anorexia nervosa (11%) [4]. Adolescents and young females with comorbid alcohol use disorders are at higher risk of developing an eating disorder or related symptoms [5]. White individuals have a higher lifelong prevalence of anorexia nervosa and binge eating disorders compared to other races/ethnicities [6].

The dysregulation of dopaminergic levels in the frontostriatal circuit can be amenable to the failure to form hedonic associations with reward stimuli [7]. When individuals with eating disorders consume normal (compared to those without eating disorders) amounts of food, it can cause a copious amount of serotonin response in the mesolimbic circuit, linking eating with the onset of dysmorphia [8]. Many studies have suggested that dysfunctions in brain structures such as the parietal cortex can be related to the perception of body image distortion; meanwhile, the striatum in the basal ganglia can be associated with altered motivation and abnormal responses to food [7]. Eating disorders can present with psychological features such as social insecurity, ineffectiveness, and poor impulse regulation. These patients experience a higher sense of loneliness, failure, and helplessness. This frame of mind predisposes an individual to a higher risk of developing depression and suicidal behaviors [9]. Bulimia nervosa and self-induced vomiting have a strong correlation with suicide attempts in children with eating disorders [10].

Compared to the general population, rates of completed suicide are higher in patients with anorexia nervosa (by 18 times) followed by those with bulimia nervosa (by seven times) [11]. A study in adult females found that those with any type of eating disorder had higher rates of suicide attempts, but more importantly, those with bulimia nervosa and anorexia nervosa (binge–purge subtype) had a higher prevalence of comorbid psychiatric disorders and suicidality [12]. As per a few longitudinal studies, the standardized mortality ratio is highest in anorexia nervosa, followed by binge eating disorder and eating disorder unspecified, and lowest in bulimia nervosa [13,14].

A meta-analysis found that eating disorders were associated with significantly increased risk for suicide attempt by two times [15]. These patients usually start with non-suicidal self-injurious (NSSI) behaviors, such as compulsive actions (hair pulling, nail-biting, self-biting, self- pricking) and impulsive behaviors (self-cutting, scratching, burning, self-hitting), and laxative abuse, which is seen in both anorexia nervosa and bulimia nervosa [16,17]. Most patients with higher severity of eating disorders and/or other comorbid psychiatric illnesses may have a higher risk of suicidal ideation. In patients with anorexia nervosa, 3–20% of the patients attempt suicide, and a high rate of patients complete suicide attempts, with a standardized mortality ratio of 1.0 to 5.3 [16]. Though 25–35% of patients with bulimia nervosa attempt suicide, fewer patients attain completed suicide (or death) compared to those with anorexia nervosa [18].

The current literature on eating disorders and suicidal behaviors is based more on adult studies and limited to studies in adolescents [13,14,19]. We hypothesized that adolescents diagnosed with depression and comorbid anorexia nervosa may be at higher risk of completing suicide and may need more rigorous evaluation and management than their counterparts diagnosed with bulimia nervosa. Our study aims to evaluate the odds of association between suicidal ideations and attempt with comorbid eating disorders (anorexia nervosa vs. bulimia nervosa vs. eating disorder unspecified subtypes) in adolescents with major depressive disorder (MDD).

## 2. Materials and Methods

### 2.1. Study Sample

Ours was a cross-sectional retrospective study that included adolescent patients (age, 12–18 years) with hospital discharges obtained from the nationwide inpatient sample (NIS, 2012–2014). The NIS is a database of hospital inpatient stays compiled from billing data across the US as part of the healthcare cost and utilization project (HCUP). The HCUP is the largest inpatient healthcare utilization database in the US and includes approximately 20% of discharges from non-federal acute care hospitals [20].

### 2.2. Inclusion and Exclusion Criteria

We analyzed a total sample of 122,020 participants. All adolescents hospitalized in psychiatric inpatient units and with a primary discharge diagnosis of MDD were included in this study, out of which 1675 (1.37%) had comorbid eating disorders. A higher proportion of adolescents with eating disorders were white (74.9% vs. 65.3%) and female (91% vs. 72.8%). The sample was sub-grouped based on the co-diagnosis of eating disorders, and the eating disorders cohort comprised patients with anorexia nervosa (N = 240, 14.3%), bulimia nervosa (N = 300, 17.9%), and eating disorder unspecified (N = 1135, 67.8%). Eating disorder unspecified refers to clinical presentations of symptoms characteristic of an eating disorder that cause clinically significant distress or impairment but do not meet the full diagnostic criteria for any of the eating disorders as per the diagnostic and statistical manual of mental disorders (DSM-IV) [21].

We excluded patients below 12 and above 18 years of age, as our focus sample was adolescents. Hospitalizations in the psychiatric inpatient units for primary management of other psychiatric conditions such as anxiety disorder, disruptive behavior disorders, mood disorders, and psychotic disorders were excluded.

### 2.3. Variables

Sociodemographic variables including age, sex, and ethnicity were obtained from the NIS [22]. Suicidal behaviors including suicidal ideation (V62.84) and suicide and self-inflicted injury (E950.XX–E959. XX) were characterized based on the international classification of diseases, ninth revision (ICD-9) diagnosis codes and were identified in current hospitalization [23]. ICD-9 diagnosis codes were used to identify comorbid alcohol abuse (291.0–291.3, 291.5, 291.8, 291.81, 281.82, 291.89, 291.9, 303.00–303.93, 305.00–305.03) and drug abuse (292.0, 292.82–292.89, 292.9, 304.00–304.93, 305.20–305.93, 648.30–648.34) [24].

### 2.4. Statistical Analysis

In all analyses, we used discharge-level sampling weights provided by the NIS data to report national estimates. We compared the distributions of sociodemographics and comorbidities between adolescents with and without eating disorders by performing the Pearson’s chi-square test. We calculated the adjusted odds ratio (aOR) and 95% confidence interval (CI) using a binomial logistic regression model with a priori confounders including age (continuous), sex, and ethnicity. We performed analyses for associations of eating disorders with suicidal ideations and suicide attempts. All analyses were conducted using SPSS version 26.0 and statistical significance was set at a two-sided *p* < 0.01.

### 2.5. Ethical Approval

The NIS is a set of publicly available de-identified data with the protection of patients, physicians, and hospital-related information; hence, we were not required to seek institution review board (IRB) permission for this study [20].

## 3. Results

Comorbid alcohol abuse was seen in a significantly higher proportion of adolescents with eating disorders (8.4% vs. 5.3% in those without eating disorders), whereas there was statistically no significant difference between the cohorts in terms of comorbid drug abuse among adolescents (14.6% vs. 15.3% in those without eating disorders) as shown in Table 1.

**Figure 1:**
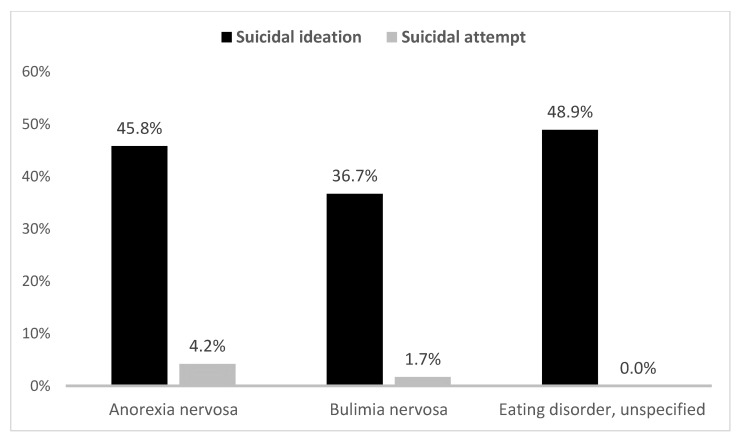
Suicidal behaviors in adolescents with major depression by eating disorders.

Suicidal ideations were seen in a significantly higher proportion of adolescents with eating disorders (46.3% vs. 14.2% in those without eating disorders), whereas a very low proportion of them had suicide attempts compared to those adolescents without eating disorders (0.9% vs. 39.4%). Suicidal ideations were seen mostly in adolescents with eating disorders unspecified (48.9%), followed by those with anorexia nervosa (45.8%) and bulimia nervosa (36.7%) as shown in Figure 1.

Eating disorders were associated with higher odds for suicidal ideations (aOR 5.36, 95% CI 4.82–5.97) compared to those without comorbid eating disorders, whereas lower odds of association were seen with suicide attempts (aOR 0.02, 95% CI 0.01–0.03). Further, the eating disorders unspecified subtype was associated with the highest odds for suicidal ideations (aOR 6.04, 95% CI 5.30–6.88), followed by anorexia nervosa (aOR 4.82, 95% CI 3.68–6.32) and bulimia nervosa (aOR 3.26, 95% CI 2.51–4.25). On the contrary, none of these subtypes of eating disorders had increased association with suicide attempts, as shown in Table 2.

## 4. Discussion

Building on the existing literature on eating disorders among adolescents, our study aimed to explore the association between eating disorder subtypes and suicidality (suicidal ideations and attempts). In our study, depressed adolescents with eating disorders had a five-to-six-times higher risk of suicidal ideations, and suicide attempts were seen in a higher proportion of depressed adolescents with anorexia nervosa (four percent) compared to those seen in those with bulimia nervosa (two percent).

Depressed adolescents with eating disorders had five-times higher overall odds for suicidal ideations but around one percent of them had suicide attempts compared to those without eating disorders. Furthermore, we found that eating disorders unspecified had the highest odds for suicidal ideations (by six times), followed by anorexia nervosa (increased by around five times) and bulimia nervosa (increased by three times).

As per a study based on the national epidemiologic survey alcohol and related conditions (NESARC-III), the lifetime prevalence of anorexia nervosa and bulimia nervosa in adolescents was 0.3% and 0.9%, respectively [3]. The prevalence of eating disorders in the adolescent inpatients with MDD in our present study was 1.37% and was seen in 91% of females. White females have a higher odds of anorexia nervosa compared to Hispanic individuals, whereas there was no difference in the odds ratio of bulimia nervosa by ethnicity [6]. This could be due to white individuals having a popular ideation of a thin body type that is media-driven and a strong sociocultural pressure to attain an “ideal body type” [25,26]. Moreover, females have a higher predisposition to developing eating disorders due to certain career choices such as modeling or cheerleading, where they strive to attain and maintain a body weight of being “Paris thin” [27]. Compared to white individuals, Hispanic and African American communities have cultural norms that are psychologically protective, in terms of body image. A larger, voluptuous body size is considered more beautiful, endorsing a larger ideal body size [28]. These communities associate dieting and weight watching to be racially incongruent behaviors [29].

There is a strong correlation between eating disorders and substance use disorders (SUDs), with a co-occurring lifetime range of 2–41% [30]. Alcohol is the most prevalent substance of choice, and other substances abused by bulimic females include cocaine and amphetamines [31]. In our study of inpatients with eating disorders, eight percent of depressed adolescents had comorbid alcohol abuse and around 15% with drug abuse. ‘Addictive personality’ traits in those diagnosed with eating disorders could be a possible cause for the higher prevalence of alcohol and drug use in this at-risk population [32].

Eating disorders are closely related to other psychiatric illnesses, and suicidal ideation and/or attempts. We found that that the adolescents with comorbid overall eating disorders (by five times), further grouped by bulimia nervosa (by three times), anorexia nervosa (by five times), and eating disorders unspecified (by six times), are associated with increased odds for suicidal ideations but had lower odds of suicide attempts when compared to their counterparts without eating disorders. Interestingly, the same pattern was seen in a large-scale longitudinal study in the adult population with eating disorders. Their crude mortality rates associated with eating disorders were eating disorders unspecified (5.2%), followed by anorexia nervosa (4%) and bulimia nervosa (3.9%) [33]; moreover, another study reported a higher standardized mortality ratio among adults with eating disorders unspecified [13]. In a study by Swanson et al. (2011), the lifetime prevalence of suicidal ideations was 61.4% in anorexia nervosa and 53% in bulimia nervosa [3], which closely relates to our findings of current suicidal ideations being higher in adolescents with anorexia nervosa (45.8%) than those with bulimia nervosa (36.7%). The prevalence of lifetime suicidal attempts in the study by Swanson et al. was higher with bulimia nervosa (35.1%) than those with anorexia nervosa (20.6%) [3]. Our study had contrary findings, with lower prevalence rates of suicide attempts in adolescents with bulimia nervosa (1.7% in bulimia nervosa and 4.2% in anorexia nervosa). However, overall eating disorders were not associated with an increased risk of suicide attempts (OR 0.069), as seen in the study by Swanson et al. [3] and further supported by our findings. A detailed evaluation and screening for concurrent depression, suicide risk assessment, and SUD should be conducted. Nutritional rehabilitation, family therapy, individual psychotherapy, and psychopharmacological treatment are treatment options [34]. This study adds to the existing (limited) literature on eating disorders among adolescents, highlighting the need to screen for eating disorders among adolescents with MDD. Those with anorexia nervosa might require more thorough evaluation due to the high risk of associated suicidality and suicide attempts. Among all subtypes, the eating disorders unspecified subtype had the highest rates for suicide ideation, and so further research that explains this finding is needed.

The results of this study should be considered with the following limitations. The NIS data are reliant on the selection of patients based on ICD-9 diagnosis codes, which may be subject to bias from underreporting or overreporting of MDD and comorbid eating disorders and/or suicidal behaviors. Demographic differences in the prevalence of eating disorder across age, sex, and ethnicity that were observed in this study were based on differences in the diagnosis of an eating disorder. Next, this was a cross-sectional study due to which odds of association between eating disorders and suicidal behaviors did not imply causal association, and we were not able to make any distinction between fatal and non-fatal suicide attempts. Nevertheless, despite these limitations, the NIS is a reliable set of data and provides a population-based national illustration of disease associations, and the large sample size ensures that it is satisfactorily powered to distinguish any differences.

## 5. Conclusions

Adolescents with MDD and comorbid eating disorders had five-times increased odds of suicidal ideations but lower odds of a suicide attempt compared to depressed adolescents without eating disorders. Among eating disorders, those with anorexia nervosa are at higher odds of suicidal ideations (by 4.8 times) than seen with bulimia nervosa (by 3.2 times). Self-harm/injurious behaviors are early signs of possible suicide ideations in these patients and require further evaluation. A collaborative care model can be implemented to screen adolescents for eating disorders during annual health check-ups to improve the early diagnosis and management of adolescents with eating disorders and improve their quality of life.

## Figures and Tables

**Table 1:** Distribution of adolescents with major depression.

Variable	Eating Disorders (−)		Eating Disorders (+)		*p*-Value
	N	%	N	%	
Inpatients	120,345		1675		-
Mean age (SD)	14.8 (1.53)		15.1 (1.42)		<0.001
Female	87,660	72.8	1525	91.0	<0.001
Race					
White	67,545	65.3	1045	74.9	<0.001
Black	11,515	11.1	110	7.9	
Hispanic	15,530	15.0	120	8.6	
Others	8825	8.5	120	8.6	
Comorbidities					
Alcohol abuse	6435	5.3	140	8.4	<0.001
Drug abuse	18,400	15.3	245	14.6	0.454
Suicidal behavior					
Ideation	17,065	14.2	775	46.3	<0.001
Attempt	47,420	39.4	15	0.9	

The proportion of major depression patients with versus without eating disorders was obtained using cross-tabulation and the Pearson’s chi-square (χ^2^) test. Significant *p* values ≤ 0.01 at 95% confidence interval. SD: standard deviation.

**Table 2:** Odds ratio of suicidality in adolescents with major depression.

Eating Disorders	Suicidal Ideation/Attempt		Suicidal Ideation		Suicidal Attempt	
	aOR (95% CI)	*p*-Value	aOR (95% CI)	*p*-Value	aOR (95% CI)	*p*-Value
Overall	0.77 (0.69–0.85)	<0.001	5.36 (4.82–5.97)	<0.001	0.02 (0.01–0.03)	<0.001
Anorexia nervosa	0.71 (0.55–0.93)	0.014	4.82 (3.68–6.32)	<0.001	0.08 (0.04–0.14)	<0.001
Bulimia nervosa	0.53 (0.41–0.69)	<0.001	3.26 (2.51–4.25)	<0.001	0.03 (0.01–0.08)	<0.001
Unspecified	0.86 (0.76–0.98)	0.024	6.04 (5.30–6.88)	<0.001	<0.001	0.987

Odds ratio generated by binomial logistic regression model and was adjusted for age, sex, and race. Significant *p* values ≤ 0.01 at 95% confidence interval. aOR: adjusted odds ratio; CI: confidence interval.

## Data Availability

Data used in this study is available from https://www.hcup-us.ahrq.gov/nisoverview.jsp (accessed on 15 March 2021).
